# Supplementary material for: Retention and Transport of Nanoplastics with Different Surface Functionalities in a Sand Filtration System
Source: Nanomaterials (Basel). 2023 Dec 21;14(1):32. doi: 10.3390/nano14010032 (PMC11326042; doi:10.3390/nano14010032)
Supplement: Supplementary file 1 [file nanomaterials-14-00032-s001.zip › nanomaterials-2778092-supplementary.pdf]

Supplementary Materials

Retention and Transport of Nanoplastics with Different Surface Functionalities in a Sand Filtration System

Hande Okutan [1,2,3], Gabriela Hul [2], Serge Stoll [2] and Philippe Le Coustumer [1,4,5,*]

[1] Ecole Doctorale, Sciences et Technologies, Université de Bordeaux Montaigne, 33607 Pessac, France; hande-mahide.okutan@etu.u-bordeaux-montaigne.fr
[2] Department F.-A. Forel for Environmental and Aquatic Sciences, University of Geneva, 1205 Geneva, Switzerland; gabriela.hul@unige.ch (G.H.); serge.stoll@unige.ch (S.S.)
[3] Department of Geological Engineering, Mugla Sitki Kocman University, Mugla 48000, Türkiye
[4] Earth Sciences Department, Université de Bordeaux, 33615 Pessac, France
[5] Bordeaux Imaging Center, Université de Bordeaux, CNRS-UAR3420–INSERM US4, 33000 Bordeaux, France
* Correspondence: philippe.le-coustumer@u-bordeaux.fr

S1. Sodium Dodecyl Sulfate (SDS) Critical Concentration Determination

A critical SDS concentration, for which all surfactant molecules present in the solution are adsorbed on the nanoplastics surface, was estimated for different polystyrene amidine latex nanoplastics (NPLs) concentrations. A solution of given polystyrene amidine latex NPLs concentration and of given SDS concentration was prepared and mixed using an orbital shaker (IKA KS 260, Germany) for at least 15 min. The z-average hydrodynamic diameter and the zeta (ζ) potential of NPLs were subsequently measured using ZetaSizer Nano ZS. The SDS critical concentration corresponds to the first concentration for which ζ potential of NPLs stops changing and has roughly the same value. A formation of a plateau of ζ potential values for 100 mg/L, 500 mg/L, and 1000 mg/L PS NPLs was observed for 300 mg/L, 400 mg/L, and 500 mg/L SDS concentration, respectively (Figure S1). These concentrations were subsequently used to prepare SDS-coated PS amidine latex NPLs.

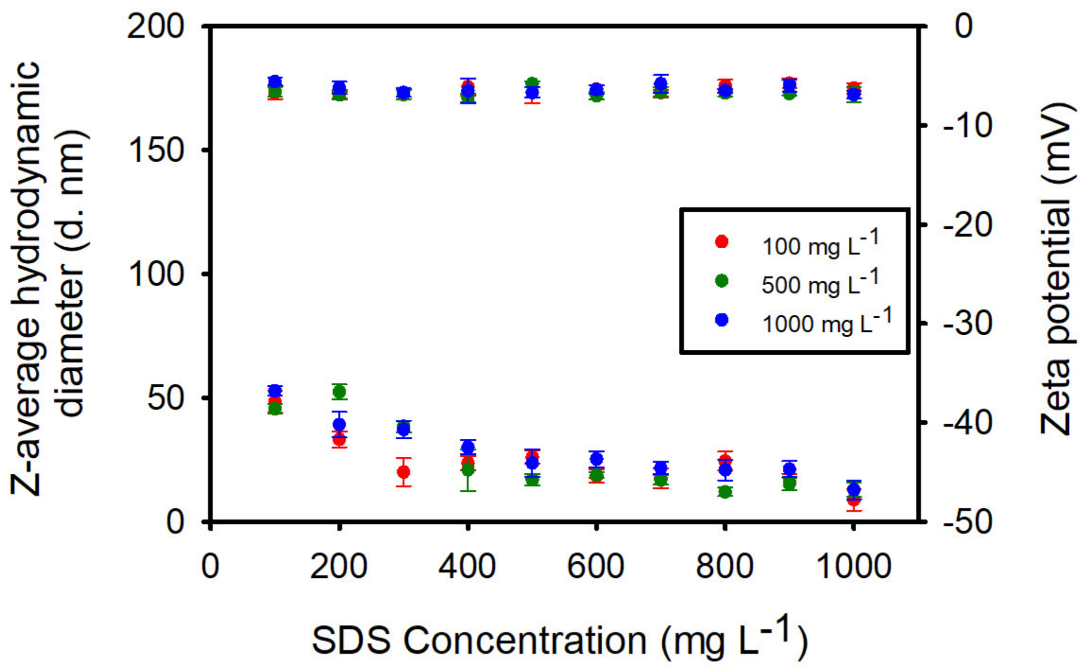

Figure S1. SDS critical concentrations for different polystyrene amidine latex nanoplastics concentrations. Experimental conditions: ultrapure water.

S2. Sand Grains Characteristics Provided by the Industrial Services of Geneva (SIG)

Table S1. Sand grains characteristics determined with Camsizer (Retsch). Sand grains belong to 11 granulometric classes and mainly have sizes comprised between 2.38 and 2.83 mm, 2.83 and 3.36 mm as well as between 3.36 and 4.00 mm. They are of ellipsoidal shape, with an average width-to-length ratio equal to 0.655, and their surfaces are rather heterogenous, with an average roughness equal to 0.826 and an average convexity close to 1.

**Grain Size (mm)**	**Volume Proportion of Particles in the Range (%)**	**Cumulative Distribution (%)**	**Sphericity**	**Aspect Ratio**	**Convexity**
0 - 0.75	0.05	0.05	0.785	0.660	0.979
0.75 – 1	0.00	0.05	0.806	0.581	0.992
1 – 1.2	0.01	0.06	0.567	0.328	0.979
1.2 – 1.5	0.07	0.13	0.577	0.310	0.982
1.5 – 2	1.54	1.67	0.708	0.437	0.986
2 – 2.38	7.26	8.93	0.774	0.529	0.988
2.38 – 2.83	24.74	33.67	0.823	0.622	0.989
2.83 – 3.36	40.64	74.31	0.845	0.692	0.989
3.36 – 4	22.98	97.29	0.848	0.728	0.987
4 – 4.76	2.67	99.96	0.826	0.761	0.982
> 4.76	0.04	100	0.654	0.742	0.956

S3. Porosity Calculations of Sand

The porosity properties of quartz sand were investigated with a fluorescein tracer experiment and its mathematical equation was implemented with 1D Advection and

Dispersion Equations which are described for the solute transport in a flow channel as follows:

$$\frac{\partial C}{\partial t} = D_L \frac{\partial^2 C}{\partial x^2} - u \frac{\partial C}{\partial x} \qquad (2)$$

Where C is solute outflow concentration [ML^{-3}], t is time variable [T], x is the distance [L], D_L [L^2T^{-1}] and u [LT^{-1}] are the dispersion coefficient and mean velocity, respectively [1].

Its analytical solution for instantaneous solute injection in a semi-infinite medium was expressed by Kreft and Zuber [2] and used for porosity calculations while doing inverse modeling between experimental and model values.

The used analytical equation is for instantaneous solute injection in a semi-infinite medium follows:

$$C_i(x,t) = \frac{M_0\, x}{A\, n\, u_x \sqrt{4\pi D_x t^3}} \exp\left(-\frac{(x - u_x t)^2}{4 D_x t}\right) \qquad (1)$$

where Ci is Concentration at the sampling point [ML^{-3}], M_0 is recovered mass [M], x is the distance between the sampling point and the injection point [L] u is the mean velocity [LT^{-1}], DL is dispersion coefficient [L^2T^{-1}], t is time [T], A is the Cross-sectional area of column [L^3] and n is effective porosity [-].

The fluorescein sodium salt concentration was measured in 460 nm with spectrophotometer both establishing calibration curve and column experiment effluents. The correlation coefficient, intercept and slope are found as 0.99, -0.007 and 0.076, respectively.

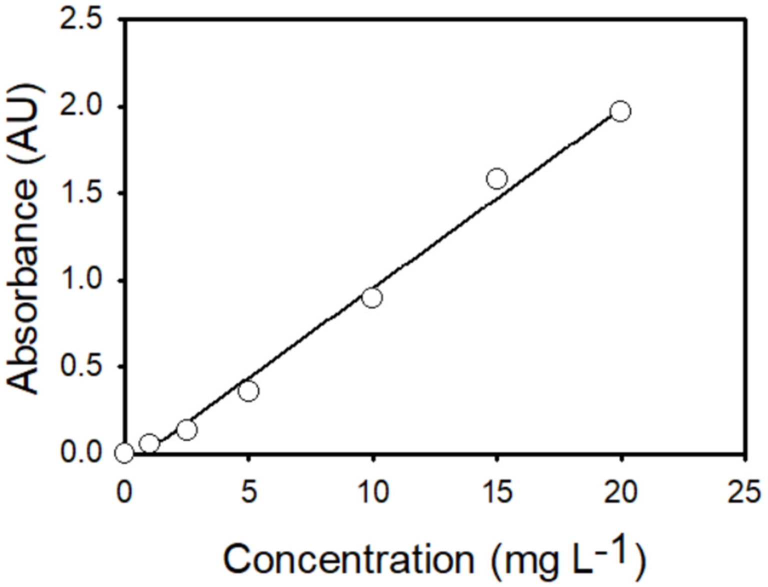

Figure S2. The calibration curve which has relation between absorbance and concentration of fluorescein sodium salt.

The porosity was calculated as 0.41 ± 0.1 by fitting experimental breakthrough curve to model breakthrough curve while manipulating porosity, mean velocity, and dispersion coefficient values with solver extension in Excel.

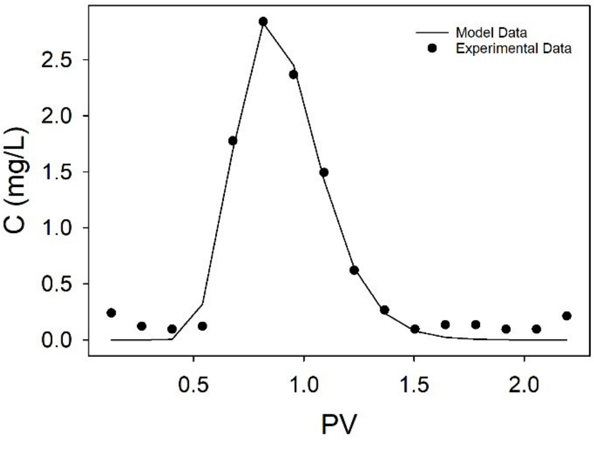

Figure S3. The experimental breakthrough curve and Model breakthrough curve after fitting procedure. The dye tracer experiment is duplicated.

S4. Turbidity Measurements

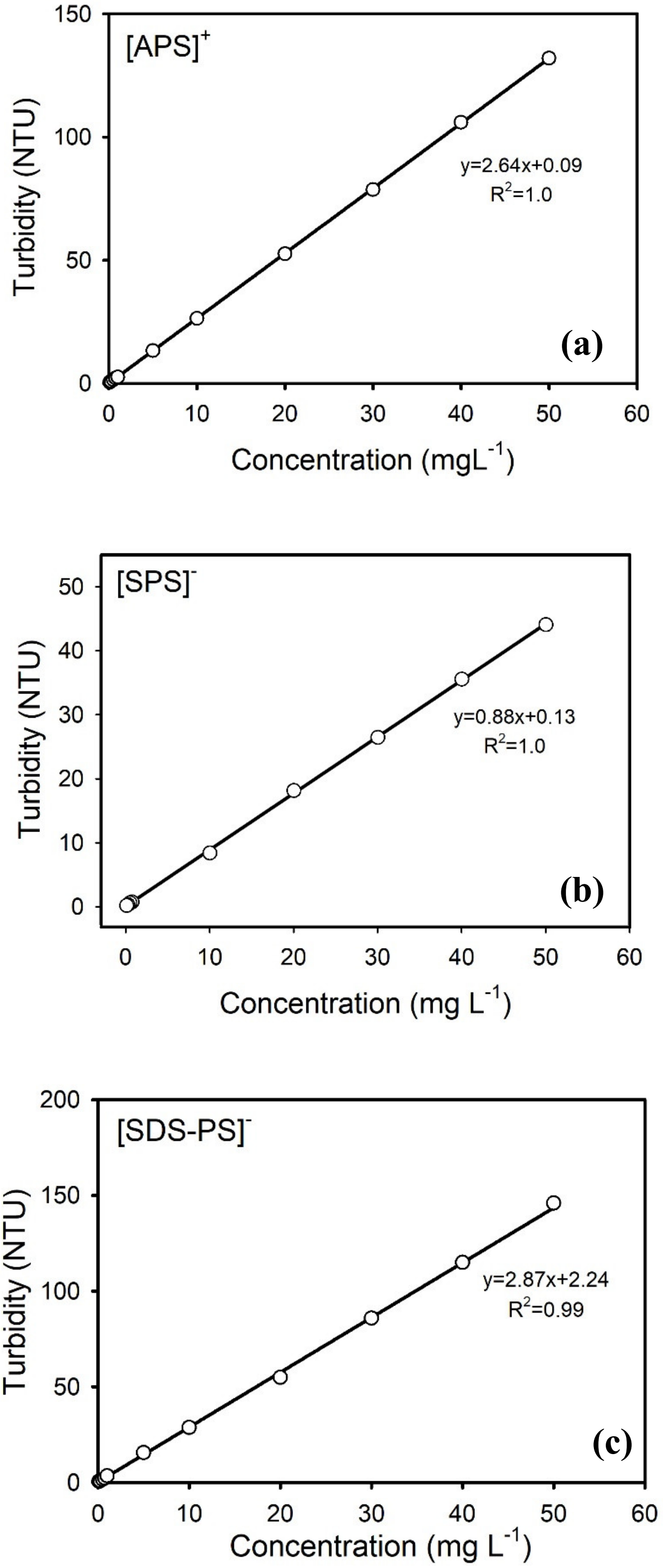

Figure S4. The calibration curves which presents relation the concentration and turbidity measurements for $[A\text{-}PS]^+$ (a), $[S\text{-}PS]^-$ (b) and $[SDS\text{-}A\text{-}PS]^-$ (c).

S5. Preparation of SEM/TEM Samples

3.1. Preparation of solid samples for SEM imaging

A thin layer of a special glue was first spread out on the 5 × 5 mm silicon wafer (G3390, Agar Scientific) attached to an aluminum stub with a diameter of 12.7 mm. Then, a few sand grains sampled from the filtration column were placed and delicately embedded in the glue up to half of their height. Such prepared samples were air-dried for at least 48 hours. Prior to analysis, they were also covered with 10 nm gold coating.

3.2. Preparation of liquid samples for TEM imaging

The carbon and holey carbon support with copper grids was used to deposit 10 μL (one drop) of NPLs dispersions or effluent samples. The grids were dried under a glass cover with infrared lamp and introduced to the microscope (Hitachi H7650).

S6. Column Experiments

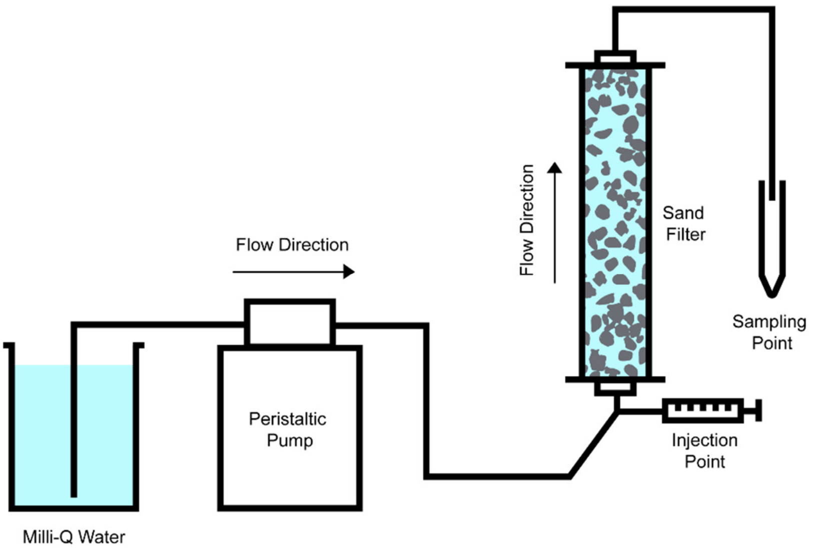

Figure S5. The schematic illustration of used experimental setup.

S7. Characterization of NPLs with Different Surface Functionalities

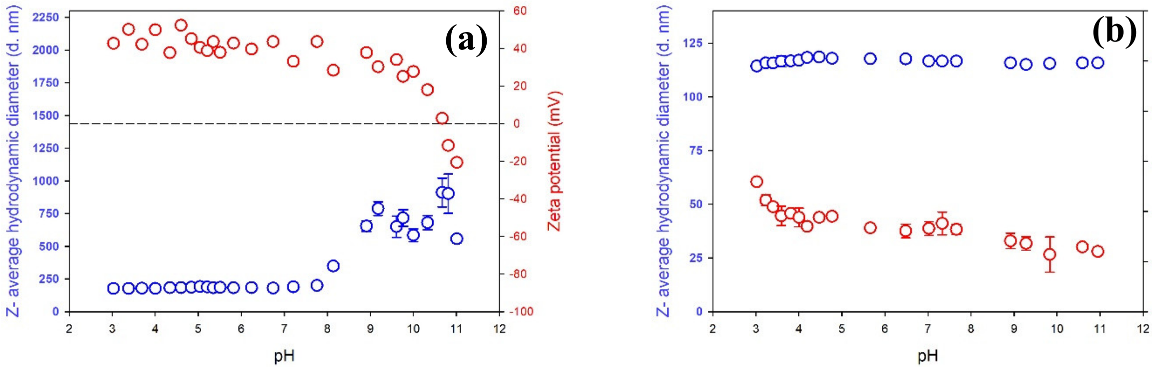

Figure S6. Size and ζ potential titration curves for (**a**) $[A\text{-}PS]^+$ and (**b**) $[S\text{-}PS]^-$.

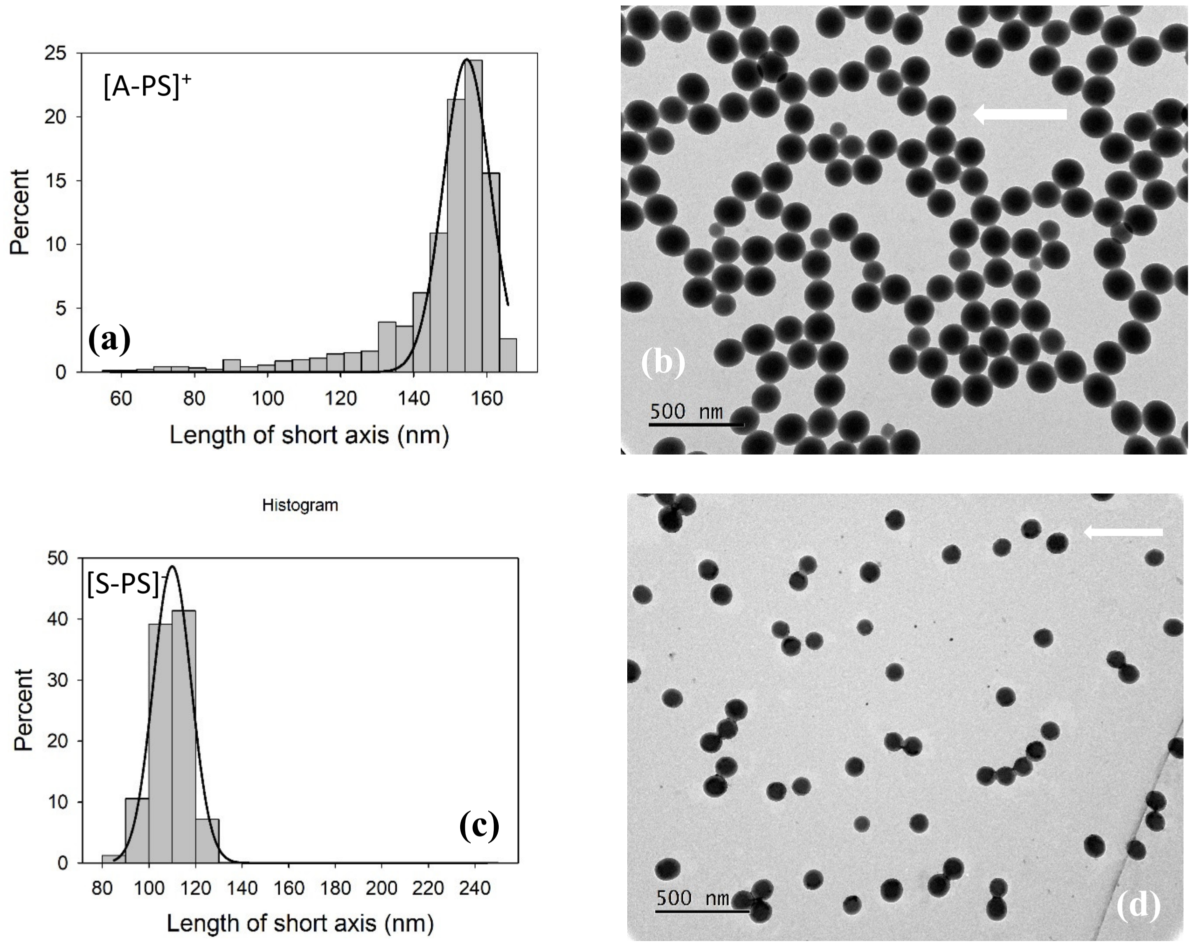

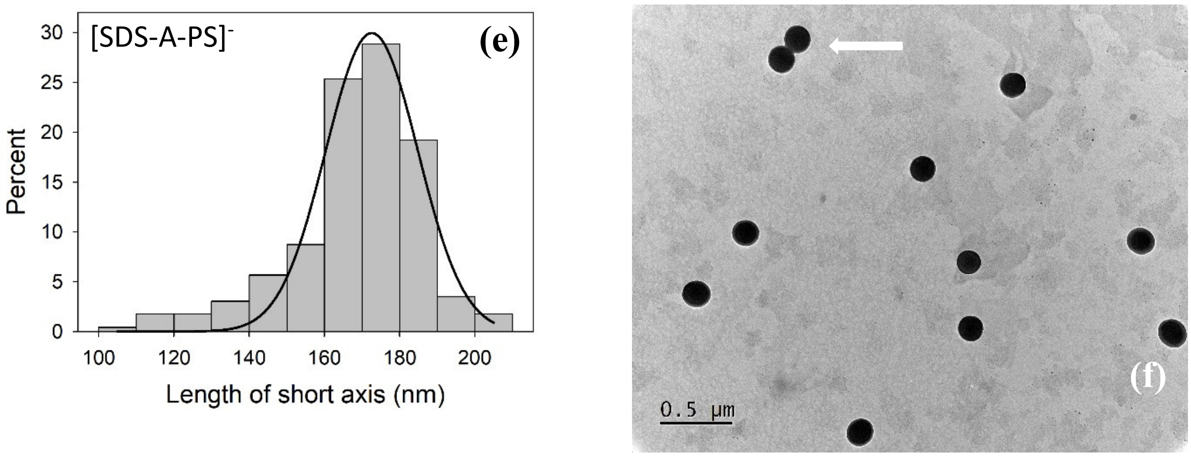

Figure S7. (**a**,**b**) [A-PS] + (**c**,**d**) [S-PS]- (**e**,**f**) [SDS-A-PS]- NPLs (white arrows) size distribution which was measured from TEM micrography (pH = 3 ± 0.1)

Table S2. Z-average hydrodynamic diameter (nm) and zeta potential (mV) measurements of NPLs (pH = 3 ± 0.1).

Amidine PS		Sulfate PS		SDS Coated PS	
nm	mV	nm	mV	nm	mV
177 ± 4	43 ± 2	114 ± 1	-34 ± 0	172 ± 1	-44 ± 0

Table S3. Statistical parameters for NPLs from the TEM image measurements in "nm" unit (pH = 3 ± 0.1).

**Type of NPLs**	**Size of Population**	**Mean**	**σ**	**Max**	**Min**	**Median**	**25 %**	**75 %**
Amidine PS	917	147	17	168	55	152	144	157
Sulfate PS	964	109	9	244	83	109	104	114
SDS Coated PS	229	168	17	204	105	170	161	179

S8. NPLs Transport Behavior in Quartz Sand Columns

Table S4. Summary of injection conditions, maximum effluent concentrations (MEC), and retained mass values for intermediate concentration conditions.

Type	Injection Concentration (mg/L)	MEC (mg/L)	Retained Mass (%)
$[A\text{-}PS]^{+}$	150	0.4 ± 0.1	79 ± 4
	200	0.4 ± 0.1	84 ± 5
$[S\text{-}PS]^{-}$	150	1.2 ± 0.1	15 ± 4
	200	1.7 ± 0.1	17 ± 6

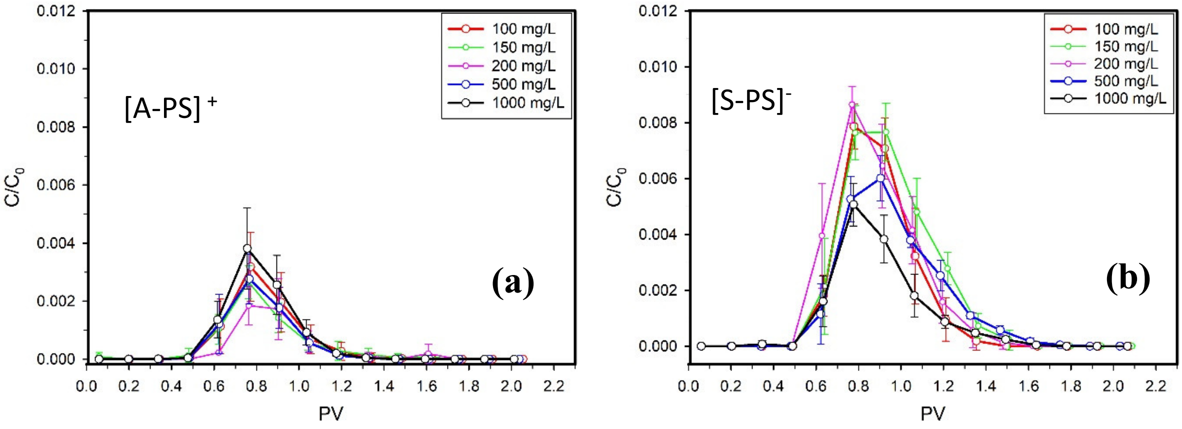

Figure S8. Observed breakthrough curves for 100 mg/L, 150 mg/L, 200 mg/L, 500 mg/L and 1000 mg/L injection concentration (C_0) are shown for (**a**) $[APS]^{+}$ (**b**) $[SPS]^{-}$.

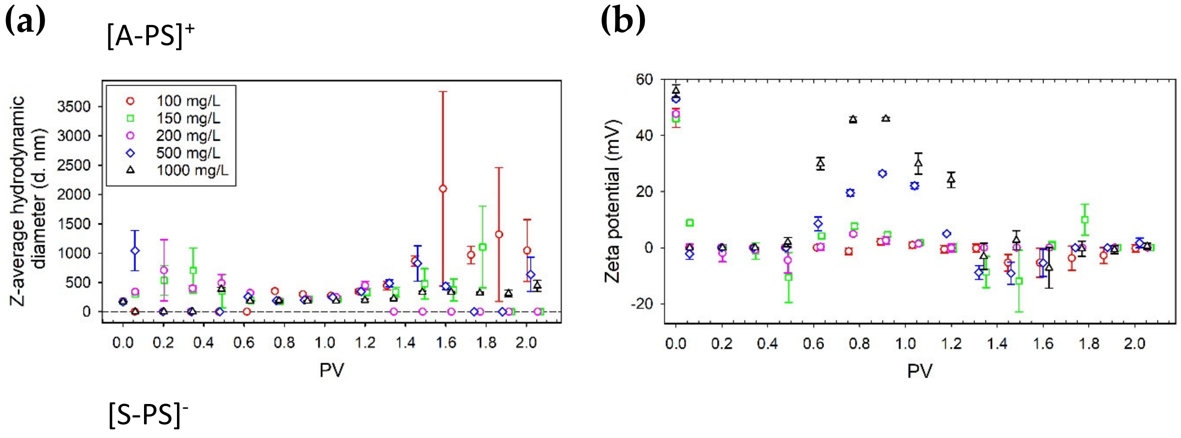

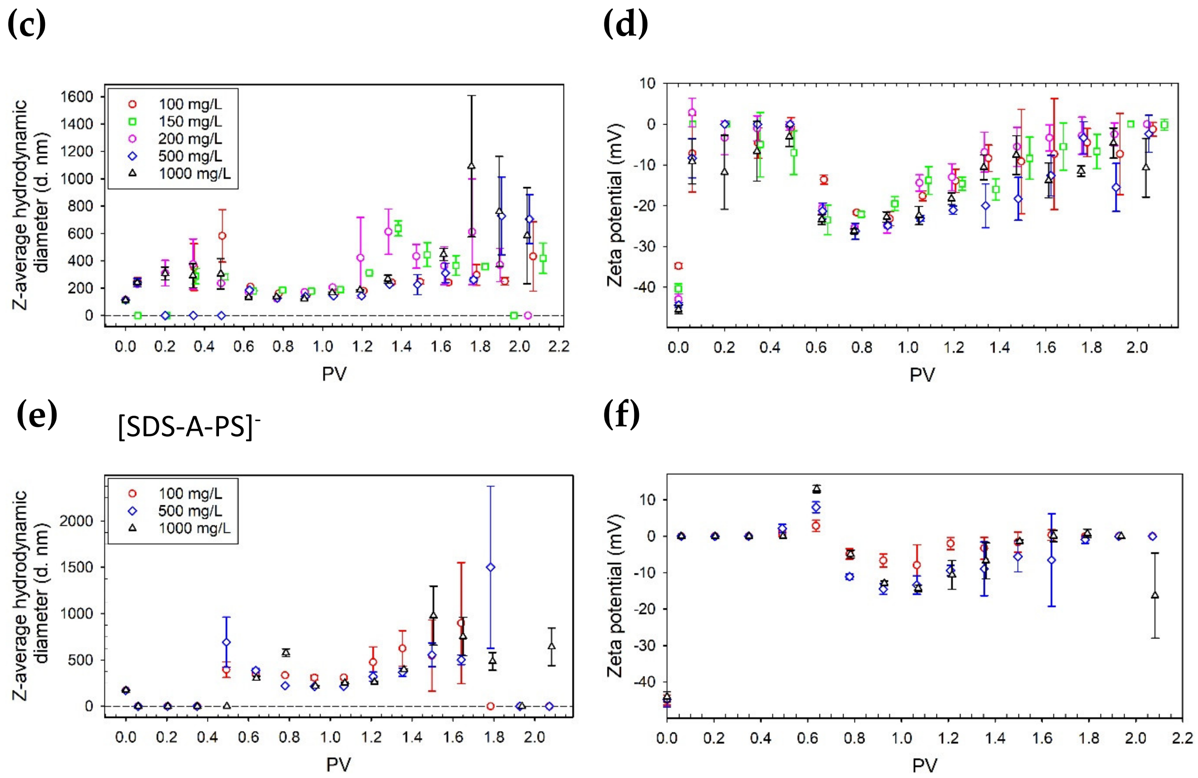

Figure S9. Collective representation of hydrodynamic diameter and ζ potential vs PV graphs of experiments performed with [A-PS]$^+$ (**a**,**b**), [S-PS]$^-$ (**c**,**d**) and [SDS-A-PS]$^-$ (**e**,**f**) for all effluents.

S9. Re-Mobilization (Desorption)

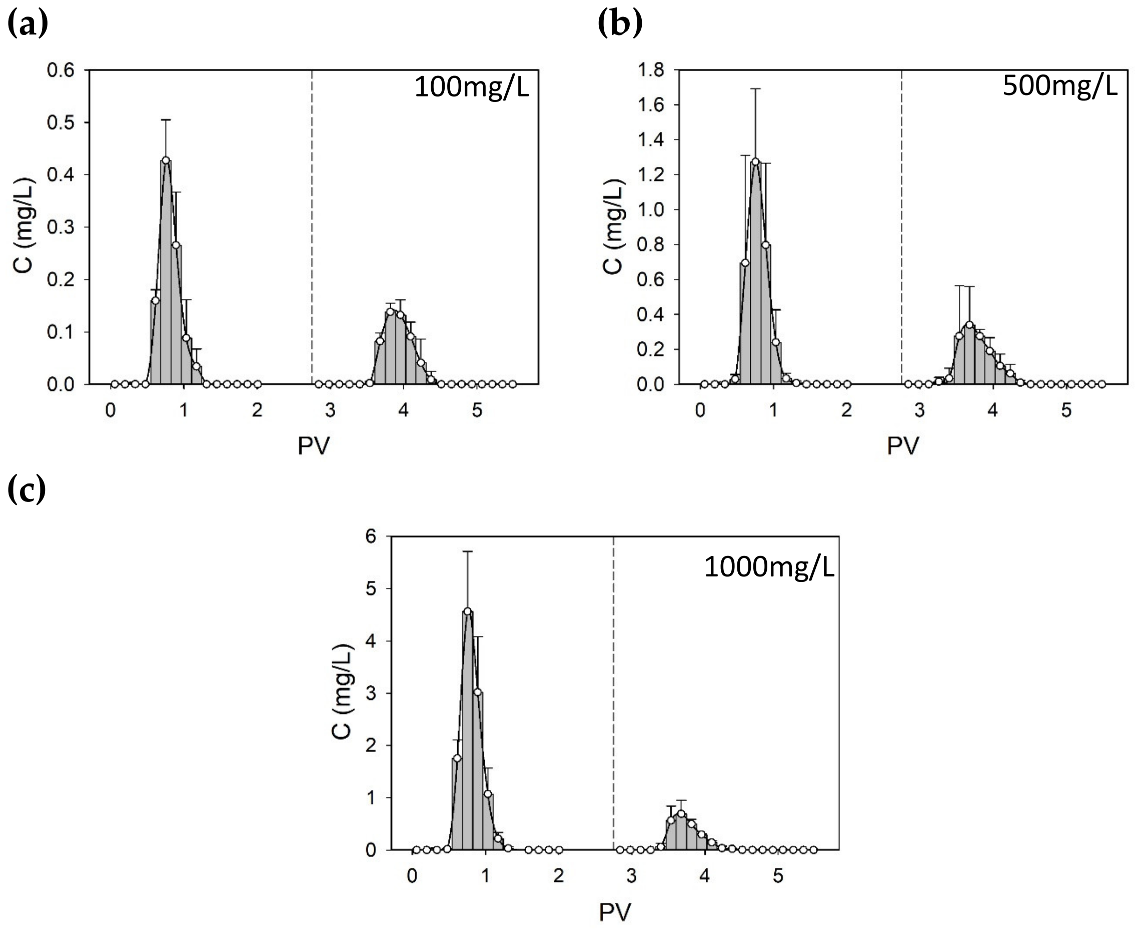

Figure S10. The breakthrough curves of adsorption and desorption experiments with 100 mg/L (**a**), 500 mg/L (**b**) and 1000 mg/L (**c**) $[A\text{-}PS]^+$ injection. The dash line represents the SDS injection.

S10. Aggregation Fractal Dimension

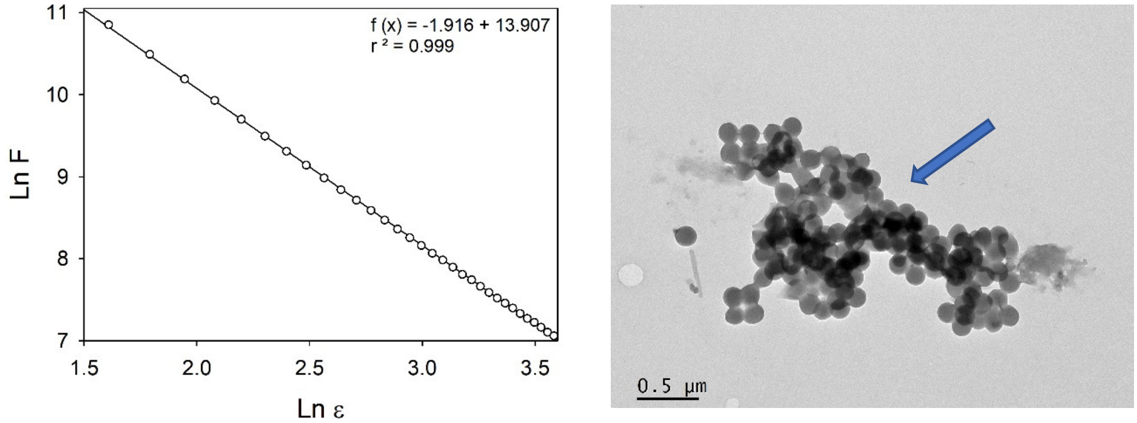

(a) (b)

Figure S11. (**a**) Log-Log pilot of X axis is the ratio of the box size to the image size and Y axis is the number of pixels counted in per box for $[A\text{-}PS]^+$ 500 mg/L injection conditions. (**b**) The TEM image of the $[A\text{-}PS]^+$ NPLs' homoaggregate (blue arrow) was used for D_f calculation.

References

1. Maloszewski, P.; Harum, T.; Benischke, R. Mathematical modelling of tracer experiments in the karst of Lurbach system. *Hydrogeologie* **1992**, *43*, 116–136.
2. Kreft, A.; Zuber, A. On the physical meaning of the dispersion equation and its solutions for different initial and boundary conditions. *Chem. Eng. Sci.* **1978**, doi:10.1016/0009-2509(78)85196-3.
